# EEG-Based Emotion Classification for Alzheimer’s Disease Patients Using Conventional Machine Learning and Recurrent Neural Network Models

**DOI:** 10.3390/s20247212

**Published:** 2020-12-16

**Authors:** Jungryul Seo, Teemu H. Laine, Gyuhwan Oh, Kyung-Ah Sohn

**Affiliations:** 1 Department of Computer Engineering, Ajou University, Suwon 16499, Korea; jrseojr@naver.com; 2 Department of Digital Media, Ajou University, Suwon 16499, Korea; teemu@ubilife.net (T.H.L.); drghoh@ajou.ac.kr (G.O.); 3 Department of Artificial Intelligence, Ajou University, Suwon 16499, Korea

**Keywords:** dementia, Alzheimer’s disease, EEG, sensor, machine learning, deep learning, emotion, classification

## Abstract

As the number of patients with Alzheimer’s disease (AD) increases, the effort needed to care for these patients increases as well. At the same time, advances in information and sensor technologies have reduced caring costs, providing a potential pathway for developing healthcare services for AD patients. For instance, if a virtual reality (VR) system can provide emotion-adaptive content, the time that AD patients spend interacting with VR content is expected to be extended, allowing caregivers to focus on other tasks. As the first step towards this goal, in this study, we develop a classification model that detects AD patients’ emotions (e.g., happy, peaceful, or bored). We first collected electroencephalography (EEG) data from 30 Korean female AD patients who watched emotion-evoking videos at a medical rehabilitation center. We applied conventional machine learning algorithms, such as a multilayer perceptron (MLP) and support vector machine, along with deep learning models of recurrent neural network (RNN) architectures. The best performance was obtained from MLP, which achieved an average accuracy of 70.97%; the RNN model’s accuracy reached only 48.18%. Our study results open a new stream of research in the field of EEG-based emotion detection for patients with neurological disorders.

## 1. Introduction

Based on the reports from Alzheimer’s Disease International (ADI), the number of Alzheimer’s disease (AD) patients has been increasing [1,2]; 46.8 million patients suffered from AD in 2015 [1], and this number increased to 50 million in 2018 [2]. Furthermore, in the 2015 ADI report [1], the number of patients with AD was predicted to reach 131.5 million in 2050; however, the predicted value was revised upward to 152 million in the 2018 ADI report [2], indicating that the number of AD patients has increased faster than experts’ expectations. Many medical researchers are investigating ways to cure Alzheimer’s disease, and many of their findings can help slow or even stop dementia’s progression; however, none cure it [2]. Given the above facts, discovering new solutions to care for AD patients is becoming critical. From the cost aspect, one trillion US dollars were spent caring for patients with the disease in 2018; however, by 2030 that figure is expected to be two trillion US dollars [2]. Caring for patients with AD often involves personal services, such as those of a nurse, family member or neighbor. Thus, the increased number of AD patients translates to increased costs, which families and governments must absorb. Furthermore, while the number of caregivers is limited, the number of AD patients is increasing, which means that the caregiver burden is increasing. Therefore, countermeasures are necessary to solve this issue.

To address these issues, we launched a project to develop a virtual reality (VR) system that can classify AD patients’ emotions and change its content based on the patients’ classification results. The purpose of the system is to increase caregiving resources’ efficiency by extending the time that patients engage with our VR system compared to other conventional VR systems. When patients are engaged with VR content, caregivers can concentrate on other tasks, which improves their efficiency. In detail, if the system recognizes an AD patient’s emotional status as bored within a certain period, it changes the VR content to something that is expected to evoke positive emotions. In the long-term, the detection of and responses to other, especially negative, emotions will also be implemented. The exact type of content to be shown can be personalized according to the preferences of the patient. By changing the VR content, we hypothesize that a bored AD patient would increase their engagement with the VR content; consequently, we expect to achieve the goal of the VR system. It is noteworthy that the adaptive VR system as described above has not yet been implemented, which is our future research objective.

To provide emotion-adaptive content, the first task in our research involves developing an emotion classification model. To classify the patients’ emotions using computing devices, among multiple possible data sources, we adopted electroencephalography (EEG) because EEG electrodes are relatively easy to install on a VR headset, and we hypothesized that this physiological signal can accurately reflect the emotional states of AD patients. At the same time, we planned to merge an EEG sensor and VR headset together physically; therefore, we selected an EEG sensor that had a suitable form for merging. In this study, we present the method we developed to train an emotion classification model; we will address the goal of extending the VR interaction through emotion-adaptive content for AD patients in a future study.

In recent years, substantial technological advances have been achieved in information technology, sensors, and the Internet of Things. Consequently, many developers and researchers have attempted to utilize these in the context of caring for AD patients. For example, Blackman et al. developed a VR system with the goal of increasing the quality of life for AD patients while decreasing the speed of their dementia progression [3]. Their system design included the ability to provide adaptive content for people with dementia; however, the adaptations were performed manually using the system controller [3]. Donovan et al. created a health care system that classified emotions from webcam videos for home-bound people with dementia and delivered the emotion classification results to caregivers and medical professionals to help provide effective tools for providing more holistic care [4]. Lin et al. designed a home-care system for people with dementia that uses radio frequency identification, a global positioning system, and a global system for mobile communication to prevent accidents and search for missing people [5]. In summary, the purposes of these previous studies were to help people with dementia in terms of medical or safety aspects [3,4,5]. However, no prior studies exist that classify the emotions of AD patients using EEG signals to improve their care.

To pursue this aim, we first conducted a literature review. To the best of our knowledge, many of the studies that classify emotions from physiological data have used young and healthy people as test subjects [6,7,8,9,10,11,12,13,14,15,16,17,18,19,20,21,22,23,24,25,26,27,28,29,30,31,32,33,34]. We also discovered several studies that utilized physiological data from patients with neurological diseases for emotion classification [35,36,37,38,39,40,41,42,43,44]; however, none of these studies focused on analyzing EEG data collected from patients with AD. To fill this research gap, we collected EEG data from 30 AD patients with dementia. During the data collection process, the patients watched videos that were intended to evoke the emotional states of happiness, peacefulness, and boredom. Based on the collected data, as an initial test, we used Weka to train several models with conventional machine learning (CML) algorithms. The results showed that the model based on a multilayer perceptron (MLP) achieved higher accuracy than did the other tested CML-based models. We also trained models with deep learning algorithms to evaluate their performance compared to the CML-based models. To utilize the sequential characteristics of time-series data, we designed recurrent neural network (RNN) models using PyTorch. To measure the models’ performances, we evaluated them via multiple iterations of five-fold cross-validation. The MLP-i model that assumes the same number of hidden states as the number of input features reached the highest average accuracy of 70.97% (maximum: 76.67%; minimum: 61.67%), whereas the best average accuracy of the RNN model reached only 48.18% (maximum: 61.67%; minimum: 35%).

The following sections describe the work conducted in this study. First, we review previous works related to this study. Second, we present our data collection methodology and equipment. Third, we explain the details related to model training. Finally, we show the trained models’ performances and conclude this study.

## 2. Background

In this section, the existing literature related to this study is reviewed. In the first subsection, we introduce medical studies that explain dementia characteristics, which are needed to design the data collection protocol for this study. In the second subsection, we summarize the previous studies that classified emotions using data collected from patients with neurological disorders.

### 2.1. Dementia

The World Health Organization (WHO) defines dementia as a syndrome that causes mental deterioration; dementia can affect memory, thinking ability, and the ability to perform everyday activities [45]. In Kim and Na’s study [46], dementia is a factor in subtypes of approximately 50 diseases, which they categorized into the following 11 groups: degenerative disorders, vascular dementia, vitamin deficiencies, endocrine and other organ failures, neoplastic diseases, chronic infections, diffuse brain damage, toxic disorders, psychiatric disorders, additional conditions in children or adolescents, and others. According to WHO [45], dementia occurs primarily in older people, and most cases of dementia are caused by Alzheimer’s (60%–70%). Other types of dementia include vascular dementia, frontotemporal dementia (FTD), and dementia with Lewy bodies. AD, FTD, and Lewy body dementia are all classified as degenerative disorders. Furthermore, the Alzheimer’s Association [47] describes the difference between AD dementia and FTD as age-related; FTD is generally diagnosed between the ages of 40 and 60, whereas the number of AD patients increases with age.

In the medical science and clinical fields, the interview-based mini-mental state examination (MMSE) has been used to screen dementia patients, as suggested by Folstein et al. [48]. It has a small practice effect; thus, it can be used multiple times on the same patient to measure differences over time [48,49]. Furthermore, modified or translated versions of the MMSE were proposed in several follow-up studies to optimize the instrument to different environments. In the Republic of Korea, several Korean versions of the MMSE (e.g., K-MMSE [50], MMSE-K [51], and SMMSE-DS [52]) have been developed. A study comparing the K-MMSE and MMSE-K [53] revealed that these two measures did not show a significant difference in reliability; however, the K-MMSE is affected by the participant’s education level, gender, and age. Thus, the study recommended using the MMSE-K [53], which consists of 30 questions. Furthermore, in a follow-up study on MMSE-K [53], Park and Kwon defined the point ranges for dementia classification as follows:

- Definite dementialess than 19.
- Questionable dementia—between 20 and 23.
- Definite non-dementia—over 24.

Regarding the behavioral characteristics of AD patients, Yoon and Park used the behaviors of dementia patients suggested by the International Psychogeriatric Association (IPA) in 1997 [54]. The major symptoms shown by monitored patients are aggression, screaming, restlessness, agitation, confusion, inappropriate behavior, sexual disinhibition, cursing, and shadowing. Regarding the correlations between dementia and emotion, Yang and Han described the following non-cognitive symptoms of dementia patients: personality changes, delusions, hallucinations, mood disorders, sleep disorders, changes in appetite, altered sexual behavior, and disturbed psychomotor activity [55]. Thus, dementia has a negative influence on the emotional states of its sufferers relative to healthy individuals. Furthermore, Na et al. explained that in a typical case, an AD patient’s brain shows damage compared to the brains of healthy individuals [56]. Therefore, the emotional processing in AD patients’ brains cannot be directly compared to that of healthy people.

### 2.2. Emotion Classification

In this section, we explain previous studies related to emotion classification. In our literature review, we searched for previous studies that classified emotions from physiological data with a specific focus on AD. The keywords were: dementia, emotion, classification, recognition, machine learning, neurological disease, and Alzheimer’s. We divided the discovered studies into two groups based on participants’ health status: healthy (see Section 2.2.1) and neurological disorders (see Section 2.2.2).

#### 2.2.1. Emotion Classification on Healthy People

To the best of our knowledge, most emotion classification studies have used data collected from healthy people [6,7,8,9,10,11,12,13,14,15,16,17,18,19,20,21,22,23,24,25,26,27,28,29,30,31,33,34,57,58,59,60,61,62,63,64,65,66,67,68,69,70,71,72,73,74,75]. Among these, 27 studies used EEG data to classify emotions [6,7,8,10,11,12,13,14,15,16,17,18,19,20,21,22,23,24,25,27,28,29,30,31,33,34,75]. As our approach uses a four-electrode EEG sensor, we focus here on studies that also used a low number of electrodes in their experiments.

Table 1 presents information on previous studies that used EEG data from up to four electrodes attached to healthy people. Singh et al. [7] and Lee et al. [11] used EEG data captured from four electrodes in their studies, whereas Takahashi [23], Shen et al. [25], Seo et al. [33,34], and Kim et al [30]. used EEG data collected from two electrodes. Regarding the positions of electrodes, other studies except Lee et al. [11] attached electrodes on participants’ foreheads [7,23,25,30,33,34]. Lee et al. [11] collected EEG data from participants’ forehead and crown of the head.

Among the studies, Lee et al. [11] trained an SVM model; however, they did not report the model accuracy. Furthermore, Kim et al. analyzed the correlation between EEG and eye tracking data by general analysis instead of training a model [30]. Regarding model performance validation approaches, Shen et al. [25], Singh et al. [7], and Lee et al. [11] did not mention the details of the approaches that they used (if any). Finally, Seo et al. [33,34] and Takahashi [23] validated their models with 5-fold cross validation and leave-one-out cross-validation (LOOCV), respectively.

The studies in Table 1 targeted at classifying healthy people’s emotions; therefore, their results are not necessarily applicable for patients with neurological disorder. However, we can conclude based on these results that EEG data captured from electrodes on the forehead have a potential for classifying human emotions. Thus, classifying emotions using EEG data captured from the forehead of an AD patient is a potential approach to investigate.

#### 2.2.2. Emotional Classification of Patients with Neurological Disorders

In our literature review, we discovered five studies that investigated classification of emotions on neurological disorder patients using physiological sensors [36,37,38,40,41]. Table 2 presents participants’ neurological disorder types by study. Only a few studies adopted patients with neurological disorders as participants; thus, we also include studies that used more than four electrodes. As the goal of this study was to classify the emotions of AD patients, we conducted a detailed analysis of these studies.

Yuvaraj et al. collected EEG data using 16 electrodes from 20 Parkinson’s patients (ten males and ten females; average age: 59.05; standard deviation: 5.64) and 20 healthy people (nine males and 11 females; average age: 58.10; standard deviation: 2.95) for their studies [36,37,38]. Kim and Na’s study [46] stated that while Parkinson’s disease is different from AD, it can also cause dementia. Yuvaraj et al. collected data from Parkinson’s patients who did not suffer from dementia [36,37,38]. Pan et al. used 30-electrode EEG data from eight patients (four males and four females; three in a vegetative state (VS) and five in a minimally conscious state (MCS); average age: 33.25; standard deviation: 13.9) and eight healthy people in their study (seven males and one female; average age: 29.2; standard deviation: 3.3) [41]. The VS and MCS patients did not have dementia. Finally, Kumfor et al. utilized data from 25 behavioral-variant FTD (bvFTD) patients (13 males and 12 females; average age: 60.7 ± 6.6), 14 semantic dementia (SD) patients (eight males and six females; average age: 64.7 ± 7.1) and 24 healthy people (12 males and 12 females; average age: 65.2 ± 6.8) [40]. According to Kim and Na’s study [46], bvFTD and SD are subtypes of dementia; however, these are not the same as dementia in AD.

Many of the previous studies that targeted data from healthy individuals referred to Russell’s circumplex model [76] to select emotions [6,19,25,30,33,34,77,78,79,80]; however, the studies that addressed patients with neurological disorders did not refer to the circumplex model [36,37,38,40,41]. For example, Kumfor et al. did not explain the reasons for selecting their target emotions [40]. Yuvaraj et al. selected six target emotions [36,37,38] that match Ekman’s six basic emotions [81]; however, they did not specifically mention Ekman’s study. Pen et al. [41] reported that they selected two emotions from Ekman’s set of basic emotions [81]. None of the studies in Table 3 involved classifying boredom or peacefulness.

Regarding the classification approaches in Table 3, none of the studies utilized deep learning algorithms for training models. Kumfor et al. identified differences in physiological data for each emotional state using statistical calculations such as ANOVA [40]. Other studies analyzed the data using machine learning methods [36,37,38,41], of which SVM was the most popular.

Yuvaraj et al. investigated ways to classify emotions from training classification models using data from both Parkinson’s patients and healthy individuals [36,37,38]. They originally used an SVM with a radial basis function (RBF) kernel and k-nearest neighbor (KNN) algorithm (K: 1–10) [37] and achieved accuracies of 86.89% (happiness), 82.56% (sadness), 79.99% (fear), 80.98% (anger), 91.27% (surprise), and 80.57% (disgust) with the SVM model [37]. In a subsequent study [36], they trained models using SVM (linear, polynomial, and RBF kernels) and fuzzy KNN (K: 3, 5, and 7) and achieved a classification accuracy of 76.90% on all the studied emotions by applying different features. Finally, their most recent study used an SVM (RBF kernel) that achieved an accuracy of 52.99% [38]. All these reported accuracy values from Yuvaraj et al.’s studies are the test results using data from patients with Parkinson’s [36,37,38]. Pan et al. also trained an SVM algorithm for their emotion classification model; however, they did not report which kernel they used [41]. They presented their results for the participants and features individually; the best average accuracy was 60.25% (maximum: 78%, minimum: 50%) [41].

Regarding the suggested models’ performance evaluation approaches, Yuvaraj et al. used 10-fold cross validation [36,37,38]. Pan et al. collected data from 16 participants by repeating the same experiments; therefore, they secured a sufficient number of samples for splitting the data into training and testing sets, the latter of which was used to evaluate the performance of the model that was trained with the former [41].

#### 2.2.3. Summary

Considering the above analysis of studies using data from patients with neurological disorders [36,37,38,40,41], none used data from AD patients with dementia. Furthermore, none of these prior studies trained deep learning algorithms as classification models. Therefore, guided by the results of our literature review, we collected physiological data from AD patients while they were exposed to certain emotion-evoking stimuli and trained classification models with both conventional machine learning algorithms and deep learning algorithms to determine the most suitable technique for emotion classification.

## 3. Data Collection

### 3.1. Participants

This study involved 30 Korean (all female) participants with dementia caused by AD who were patients at a medical rehabilitation center in the Republic of Korea. The average age of the participants was 83.9, and the standard deviation was 5.02. Table 4 presents the participants’ MMSE-K point distribution. The medical center measures the MMSE-K scores of their patients regularly; however, two patients had either refused to take the MMSE-K test or had not received it yet; however, according to clinical assessments, they were considered to be AD patients with dementia. Furthermore, one participant had normal MMSE-K scores in her medical record; however, her physician diagnosed her as having dementia as well.

To ensure safe and ethical research procedures and data handling, we received ethical approval for our experiment from the medical rehabilitation center’s institutional review board (approval number EH2018-1), and all patients and their guardians signed a consent form before participating in the experiment. To ensure medical safety, specialist doctors and regular nurses were present during the experiments. Furthermore, all the staff members involved in this experiment were trained to administer aid in case an accident occurred, and an emergency cardiac defibrillator was prepared and available for use. Finally, we provided each of the participants with a gift after the experiment.

### 3.2. Target Emotions

**Figure 1:**
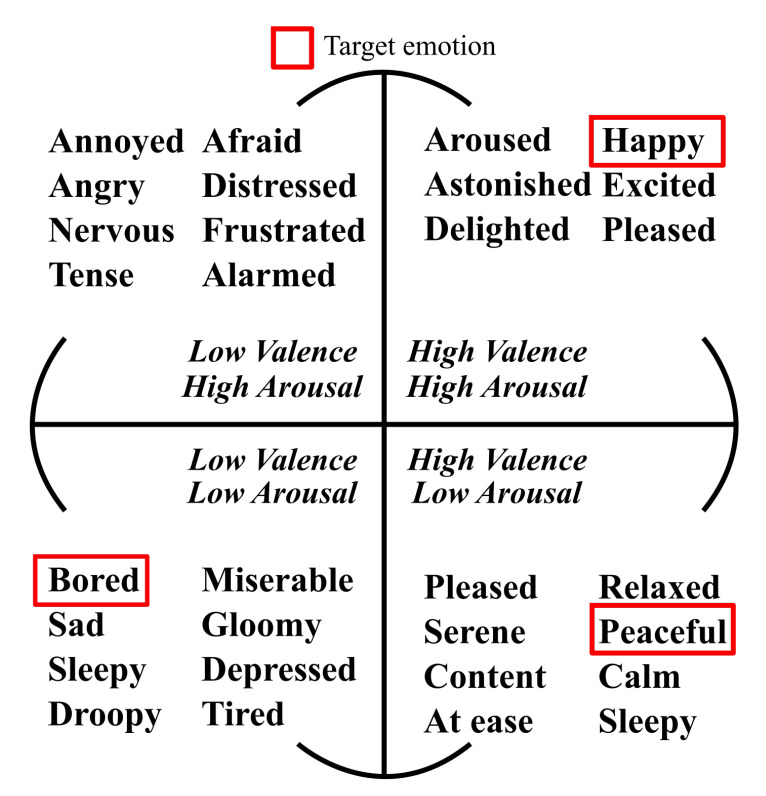
Russell’s circumplex model.

We selected the target emotions for this study by referring to Russell’s circumplex model (Figure 1). We also solicited the opinion of a physician at the center regarding emotion selection to ensure the safety of patients with AD. Based on the feedback, we selected happiness, boredom, and peacefulness as the target emotions. We did not choose an emotion from the second quadrant of the circumplex model because the doctor advised against it, as those emotions could cause shock, cardiac arrest, or other medical problems in the participants.

### 3.3. Stimuli

**Figure 2:**
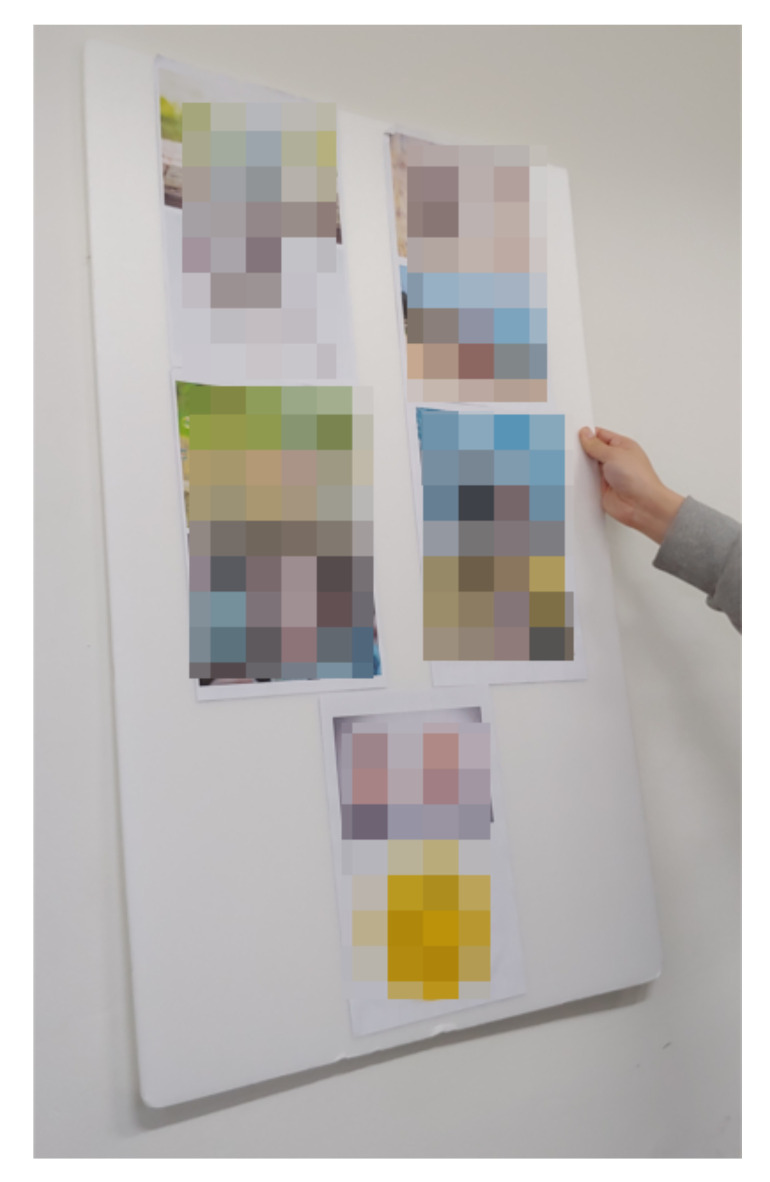
Image panel (happiness).

Considering the characteristics of people with dementia (See Section 2.1) and the comments of the physician in the medical rehabilitation center, we hypothesized that the participants may respond differently to emotion-evoking stimuli compared to healthy individuals. Thus, we prepared a stimuli selection experiment before data collection. First, we preselected 30 images (10 images per target emotion). These images were collected from the web and the International Affective Picture System (IAPS) [82]. We printed the images, attached the appropriate images to separate panels for each emotion (see Figure 2), showed the panels to the patients with AD at the medical rehabilitation center, and then interviewed the participants. The interviews were conducted in Korean. Each participant was asked to select two images that made them experience the target emotions. To avoid instances in which the subjects remembered the pictures, we allowed approximately one month to elapse between the date of image selection and the date of EEG data collection. This gap allowed the patients with AD sufficient time to forget the images.

Based on the interview results, we created a video for each target emotion consisting of five selected images. Based on the image selections made by patients with AD during the interviews, we selected the top five images. Furthermore, we added suitable sound effects to increase the emotion-evoking efficiency. For instance, we played a cat sound when a cat image was presented on the video. We showed each image for 18 s; therefore, the duration of each video was 90 s.

### 3.4. Sensor

**Figure 3:**
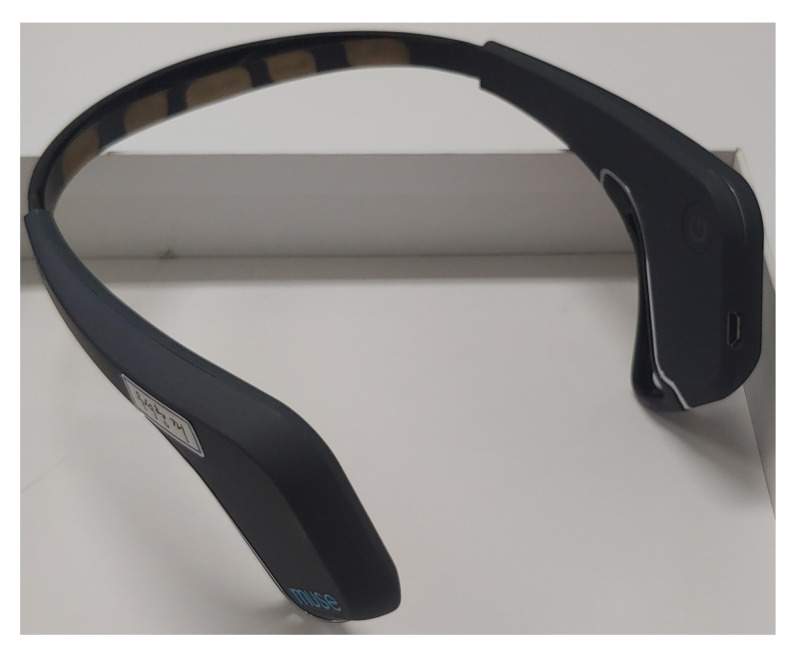
EEG sensor (Muse Brainband 2016 version).

For the data collection, we used the Muse Brainband (2016 version, see Figure 3) [83]. The sensor has four electrodes, AF7, AF8, TP9, and TP10. The electrode locations were defined by [84], and these locations are commonly used in studies that collect EEG signals [25,30,33,34,36,37,38,41,58]. We selected Muse for three reasons. First, as explained in Section 1, we considered combining an EEG sensor and a VR headset to achieve the project’s goal; therefore, multi-electrode EEG sensors were deemed to be unsuitable for integration with a VR headset from the perspective of physical design and usability. Second, our project’s target users are aged AD patients. At the moment of writing this paper, a typical VR headset has a relatively heavy weight; thus, we selected a lightweight EEG sensor to ensure that the total weight was low. The final reason was an economic issue; professional-level EEG sensors are too expensive for most end-users. For this reason, we selected the Muse headband because it was commercially available at an affordable price.

In this study, we used the data from AF7 and AF8 only because the TP9 and TP10 electrodes did not attach well to the patients’ heads during the data collection process. Each electrode had a data sampling frequency of 256 Hz. The Muse sensor collects, calculates, and outputs several types of data related to EEG. Specifically, it collects EEG data from each electrode, applies a notch filter to reduce the noise stemming from muscle movement, and automatically calculates the absolute band power (ABP) using fast Fourier transform. Furthermore, it provides several pieces of intermediate data (e.g., notch filter applied EEG data) from the raw EEG data to ABP for research purposes. The raw EEG and notch filtered EEG data are produced from each electrode at a rate of 256 Hz, and the ABP and other calculated data are produced from each electrode and frequency band at a rate of 10 Hz. The frequency bands of the Muse sensor are defined by its manufacturer as follows:

- Delta (1–4 Hz).
- Theta (4–8 Hz).
- Alpha (7.5–13 Hz).
- Beta (13–30 Hz).
- Gamma (30–44 Hz).

**Figure 4:**
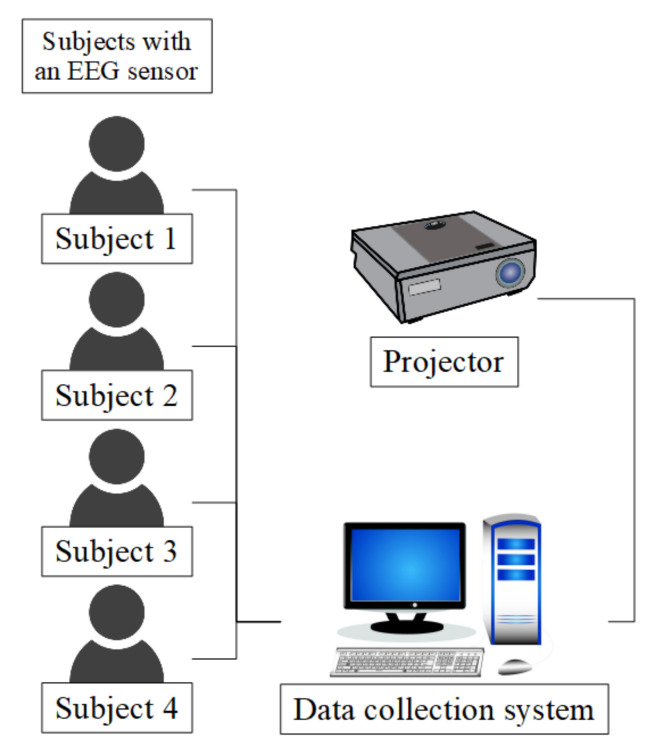
Data collection system structure.

Additionally, we placed cameras (GoPro Hero4 action cameras) with microphones in front of the participants to record their faces and voices for the purpose of identifying any unexpected variables during the experiment. The cameras were small and did not interfere with the participants while they watched the stimuli. Figure 4 shows a sketch of the data collection environment. The EEG sensors were connected to our collection system through Bluetooth 4.0. To play the stimuli for viewing by multiple participants, we utilized a projector with a speaker. The data collection system controlled the experiment automatically, displayed the statuses of all the sensors, and provided real-time information on the experiment through a display for monitoring purposes.

### 3.5. Protocol

**Figure 5:**
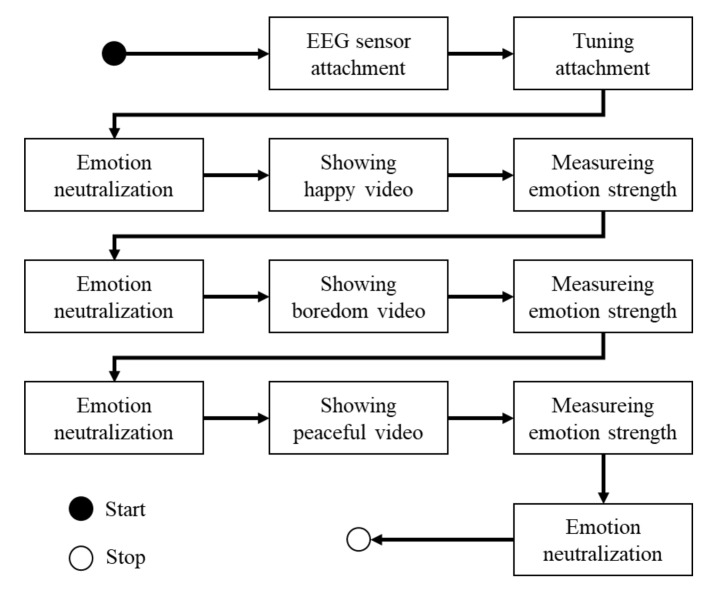
Data collection protocol.

The data collection protocol for patients with AD (Figure 5) considered the characteristics of people with dementia (see Section 2.1). In this protocol, we included three “emotion neutralization” stages before showing each emotion-evoking video clip. In the second and third neutralization stages, we asked the participants to close their eyes and breathe deeply for 30 s; however, in the first neutralization stage, we introduced ourselves to the participants to create a good mood. The emotion neutralization stages differed from the first stage based on a strong request from the physician at the medical rehabilitation center, who mentioned that many elderly people with dementia are afraid of strangers. Thus, they recommended that we introduce our research team members first to ensure that the participants felt at ease. We determined that most patients felt comfortable from their facial expressions. Finally, after showing each emotion-evoking video, we asked the participants to estimate the strength of the target emotion using the following four-point scale: (1) unknown; (2) no; (3) yes; and (4) very much. The unknown option was added to the scale because patients with AD may answer abnormally or refuse to answer.

## 4. Modeling

**Figure 6:**
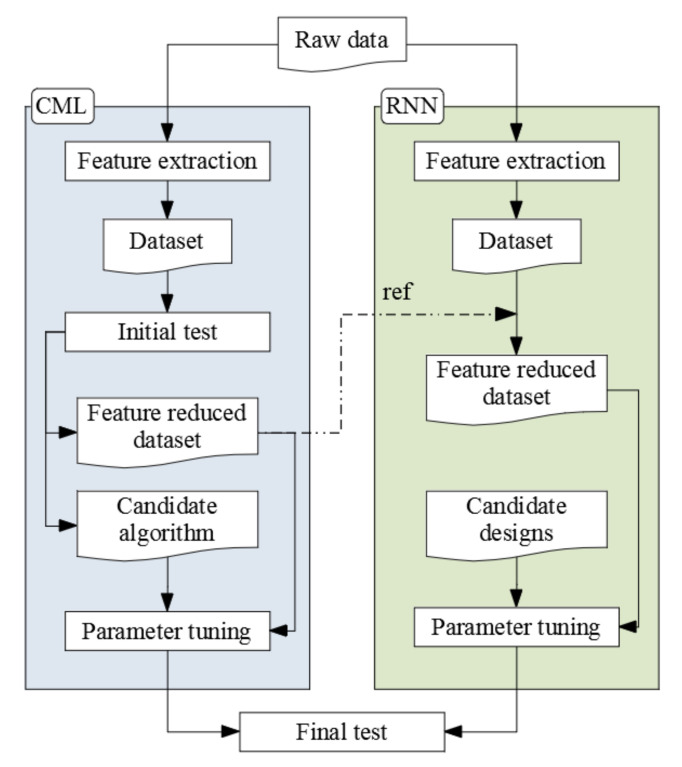
Data handling flows.

In this section, we explain the methodology to train classification models. As explained in the introduction, we trained models with conventional machine learning algorithms (CML) and deep learning (DL) algorithms. Figure 6 illustrates the data-handling flow of each training process with the CML algorithms and the RNNs. Specifically, we chose the RNN design because such models can utilize the sequential characteristics of time-series data. We discuss the feature extraction process for each CML and RNN model, the initial test processes for the CML algorithms to select candidate algorithms for parameter tuning, the RNN architecture, and the final test process for measuring the classification models trained with optimized parameters. In this study, we used a system equipped with an NVIDIA GTX 1080 Ti GPU to train the RNN models.

### 4.1. Feature Extraction

To extract features from EEG data, we adopted several window sizes to investigate the optimal size that contains the best information for classification. As explained in Section 3.3, we recorded 90 s of data for each target emotion; however, none of the records contained precisely 90 s of data. Therefore, to extract features uniformly, we set window sizes of 10, 20, 30, 40, 50, 60, 70, and 80 s. In the initial test stage, we determined the optimal window size by analyzing the results.

Regarding the extraction methods, we referred to Seo et al. [33,34] and Zheng et al. [29]. The method details are as follows:

- ABP—mean, standard deviation (std) by the frequency bands and electrodes.
- Differential entropy (DE)—by the frequency bands and electrodes.
- Rational asymmetry (RASM)—by the frequency bands.
- Differential asymmetry (DASM)—by the frequency bands.

As explained in Section 3.4, our EEG sensor produced an ABP value of 10 Hz by frequency bands and electrodes. For instance, in the 10 s raw dataset, 100 ABP values existed for each frequency band. We calculated the mean and standard deviation (std) values of these ABP values for use as features. As a result, we secured 20 features (two electrodes × five frequency bands × mean and std).

(1) $$DE=\frac{1}{2}\log2\pi e\sigma^{2}$$

(2) $$DASM=DE_{\left(left\right)}-DE_{\left(right\right)}$$

(3) $$RASM=DE_{\left(left\right)}/DE_{\left(right\right)}$$

Equations (1)–(3) were used by Seo et al. [33,34] and Zheng et al. [29] to calculate DE, DASM, and RASM, respectively; they used these equations to extract features from EEG data. In Equation (1), π and *e* are constant values, and $\sigma^{2}$ is the variance of the bandpass-filtered EEG data. We altered the bandpass filtering range based on the band definition of our EEG sensor and calculated the DE from each frequency band. Thus, we acquired ten features by calculating the DE (two electrodes × five frequency bands). As Equations (2) and (3) show, these utilize electrode position differences and DE. Many previous studies have mentioned correlations between frontal asymmetries in EEG signals and emotional status or mental tasks [8,28,85,86,87,88]. RASM and DASM utilize the frontal asymmetry of each frequency band. By calculating these features, we acquired ten features (five frequency bands × RASM and DASM). Finally, we created a dataset with 40 features and used it to train the models with CML algorithms. As 30 patients with AD watched three emotion-evoking videos, a total of 90 samples were represented in the dataset.

Regarding the feature extraction approach for RNN, we modified the CML feature extraction approach to include time characteristics in the dataset. To conduct feature extraction for the RNN dataset, we did not calculate the mean and standard deviation of ABP in a window; instead, we used the ABP value directly as a feature. Regarding the calculations of DE, RASM, and DESM, using the timestamp of each datum, we obtained the raw EEG data that were used to calculate the ABP and used that to calculate the DE, RASM, and DESM. The ABP sampling frequency was 10 Hz; therefore, in the 80 s window, 800 sequence data points were obtained for each sample. Following this modification, we prepared a dataset consisting of 30 features with 800 sequences per sample to train the RNN.

### 4.2. Initial Test

In this study, we used Weka to train the models with CML algorithms and PyTorch (version: 1.3.1; CUDA version 10.2) to train the models with the RNN. Weka is a Java-based open-source machine learning API that provides many algorithms for training models. PyTorch is a Python-based deep learning API that provides RNNs, convolution neural networks (CNNs), and other deep learning models. Specifically, the PyTorch RNN provides three types of RNN models. We investigated all the possibilities to identify the CML and RNN models with the highest performance; however, the number of algorithms in CML was too large to allow the tuning of each algorithm’s parameters to obtain the highest accuracy. Therefore, we executed an initial test to select candidate algorithms among the available CML algorithms for parameter tuning. For the RNN models, we tuned the parameters without performing an initial test.

Table 5 presents the classifier algorithm list that we used to find suitable candidate classifier algorithms. Many algorithms have parameters related to classification performance; however, investigating the performances of all the possible models trained using all possible parameter combinations for each algorithm would require an enormous amount of time. Therefore, we set many of the listed algorithm parameters to their defaults, as shown in Table 5. In the initial test, we measured the performances of the trained models using 10 iterations of five-fold cross-validation, where the random seed value for each training was different. We performed the initial test with ten iterations and summarized the test results.

According to the Weka documentation, IBk is a k-nearest neighbor (k-NN) classifier. To determine the parameters for IBk, we tested a parameter that assigns a weight for measuring the distance from other samples because we considered it to be an important parameter influencing the classification performance considering the k-NN classification methodology. The LibSVM shown in Table 5 is a support vector machine API that provides several parameters. We selected the kernel-related parameters for this investigation because the kernel is an important factor in allowing additional data dimensions in an SVM. Finally, we considered the network design for MLP because the design affects the model performance. Other parameters, such as the epoch and learning rate, are also important in achieving the best performance; however, we focused on the network design in this investigation.

In this initial test, we applied the feature selection algorithms and the wrapper subset evaluator (WSE) before training the models with each CML algorithm. The WSE automatically outputs an optimal feature subset that increases the model performance. Consequently, by applying the WSE, we could remove unnecessary features from the original dataset.

### 4.3. Design for Deep Learning

**Figure 7:**
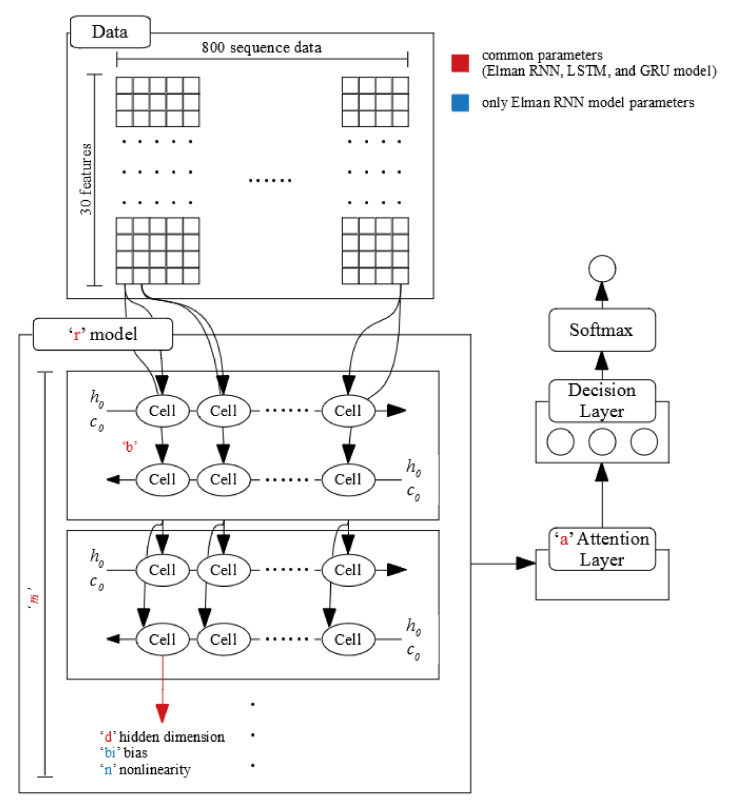
RNN model design.

Figure 7 shows the RNN model design in this study. As explained in Section 4.2, PyTorch supports the following three RNN models: Elman RNN, long short-term memory (LSTM), and gated recurrent unit (GRU) models. We investigated all the possibilities of each model type having positive or negative effects on our deep learning model. Thus, we tested all the possible cases during the parameter-tuning stage.

After the RNN layer, we attached the attention layer contained in PyTorch-NLP with the expectation that the attention layer could increase the classification performance and provide clues regarding how to interpret this model; however, we were also open to the possibility that it might have a positive effect or negative effect on our model. Therefore, we tested it for both possibilities.

The decision and softmax layers attached after the attention layer (see Figure 7) were required to obtain the classification results from the networks. The decision layer was a single fully connected layer.

### 4.4. Parameter Tuning

As described in Section 4.2, we tuned the parameters of the candidate CML algorithms. In our initial test results (see Section 5.2), we selected MLP as a candidate algorithm for tuning. The tuned parameters and their ranges were as follows:

- Learning rate: 0.001–1.000 (0.001 unit).
- Epochs: 1–500 (1 unit).
- Hidden layer design: a, t, and i (see Table 5).
- Momentum: 0.2 fix.
- Other parameters: default fix.

From this investigation, the MLP parameters most related to model performance in Weka were the learning rate, epoch, and network design. Regarding the network design, we referred to the initial test results (see Section 5.2). Following the ranges, we trained and tested 1,498,500 models with five-fold cross-validation to find the optimal parameter combinations that perform well.

As explained in Section 4.3, we designed the RNN model and then tuned the model’s elements and its parameters. Figure 7 in Section 4.3 shows the tuned parts of the model. The detailed tuning ranges were as follows:

- r: LSTM, GRU, and Elman RNN.
- m: 1,2, and 3.
- b: Single direction and bi-directional.
- d: 2, 4, 8, 16, 32, 64, 128, 256, and 512.
- a: true and false.
- bi (only using Elman RNN): True and False.
- n (only using Elman RNN): “relu” and “tanh.”
- Epochs: 1–1000 (1 unit)
- Learning rate: 0.0001 fix.

In the above list, “r” is the RNN model type, “m” is the number of stacked layers, “b” denotes whether each layer is unidirectional or bidirectional, “d” is the number of dimensions in each cell of each layer, and “a” denotes whether the attention layer is included after the RNN model group. The parameters “bi” and “n” were used only when the “r” value was Elman RNN; they indicate whether a bias weight was used and the activation function for nonlinearity in the Elman RNN cells, respectively. Specifically, if “a” is False, no attention layer is included after the RNN model in our design, and the data output by the RNN model is input directly into the decision layer. Regarding “d”, we set an exponent with two values as a value; however, we could not exceed 1024 because of the GPU memory limitations.

To avoid overfitting or underfitting issues when using artificial neural network algorithms, a suitable epoch must be selected. In MLP, we adopted the epoch that showed the highest validation accuracy and used a small learning rate. In the RNN model, we calculated the average value for each fold epoch that showed the lowest validation loss. Additionally, to ensure a fair comparison among all possible parameter combinations, we applied the same random seed value in all the training and testing.

### 4.5. Final Test

After the parameter tuning stage, we trained and tested models with each parameter-optimized algorithm as a final test. In this test, we used five-fold cross-validation and executed 1000 and 100 iterations for MLP and RNN, respectively, using different random seed values. The reason for the different number of iterations was that RNN training required lengthy times to complete 1000 iterations because of its deep learning model.

In this stage, we evaluated the models using three metrics: accuracy, AUC, and F1-score. Furthermore, using a confusion matrix, we evaluated the models’ classification performances for each target emotion. Finally, we analyzed the attention score data gathered from the attention layer in the RNN model to interpret the model’s internal workings.

## 5. Results

### 5.1. Interviews

Table 6 shows the distribution of the interview results from each stimulus. As the table shows, the stimulus videos meant to evoke happiness and peacefulness evoked the corresponding target emotions in the participants well; however, the stimulus video meant to evoke boredom was not very effective.

To interpret the results easily, we regrouped the interview results by each stimulus video. Specifically, when the interview result was “yes” or “very much”, we considered the participant to have the target emotion. Therefore, we annotated that dataset with the target emotion. For the answers of “no” or “others”, we considered the target emotion or mentally abnormal state to not have been invoked; thus, we marked those as others. We utilized the regrouped results as sample labels to train the models.

To match the purpose of this study, we removed the samples marked as others from the datasets for the following reasons: (1) For the others samples, the target emotion could not be judged as the same emotion, and (2) due to the limitations of AD patients, we did not attempt to identify which emotion was elicited by the stimuli for the samples marked as others. Table 6 shows the label distributions for the remaining samples. Specifically, 30 samples labeled others were excluded from each dataset. As a result, 60 samples marked as boredom, happiness, and peacefulness remained.

### 5.2. Initial Test

Table 7 shows the top 10 experimental setups achieving the best average accuracy in 10 iterations of five-fold cross-validation. This experiment revealed several findings. First, the MLP models with a single hidden layer (with options i, a, or t that determine the number of hidden nodes as described in Table 5) generally outperformed other algorithms. Second, 80 s was found to be an optimal window size compared to other window sizes. Finally, WSE worked well to remove unnecessary features and improve model performances. Based on these results, we tuned the MLP parameters and limited the MLP layer design parameters to a, t, and i in the parameter tuning stage.

Table 8 shows the WSE results from the initial test. These results indicate that the gamma and alpha bands are strongly correlated with the emotions of AD patients and that AF8 is more related to emotion than is AF7.

Based on the analysis, during the RNN model parameter tuning, we tested cases using both the original and the feature-reduced dataset. The feature-reduced dataset included the features AF8 Alpha DE, Gamma DASM, and Gamma RASM, but AF7 Beta and ABP mean were excluded because they were selected only once. Additionally, we limited the window size to 80 s for the RNN model parameter tuning to reduce the investigation time.

### 5.3. Parameter Tuning

Table 9 and Table 10 show the parameter tuning results for the MLP and RNN models, respectively. Each result is an optimal parameter combination that showed high classification performance and minimized overfitting issues.

The MLP accuracies in Table 9 reached 75.00%, 76.67%, and 71.67%, while the RNN accuracies of Table 10 reached only 55.00%, 60.00%, and 66.67%. Regarding the RNN model type, the Elman RNN [89], the oldest proposed RNN version, was more suitable for our dataset than more recent models such as LSTM or GRU [90], probably because it has fewer parameters to train. All the results in Table 10 are from the 80 s feature-reduced dataset, which were the best. The results of using the original dataset showed lower accuracy than those using the feature-reduced dataset for each RNN, LSTM, and GRU.

### 5.4. Final Test

Table 11 and Table 12 show the final test results and confusion matrices for each model. As Table 11 indicates, the MLP-i model performed better than the other models in terms of average accuracy and F1-score; however, its performance was slightly worse than the MLP-a model in terms of AUC. In addition, the RNN models performed worse than the MLP models. Furthermore, similar to Section 5.3, the relatively recently proposed RNN model did not classify emotion well.

As shown in Table 12, the MLP models did not classify boredom and the other target emotions. In contrast, the RNN models classified boredom better than the MLP models. Regarding the classification performance for boredom, the classification accuracies of the MLP models for boredom were 0% and 0.17%; however, the Elman RNN model achieved 44%.

**Figure 8:**
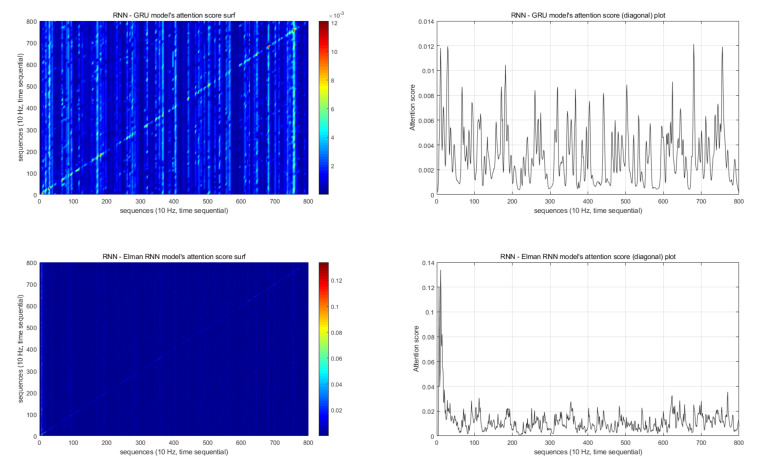
Attention scores.

Figure 8 illustrates the attention score results of the RNN models. Regarding the attention score of the LSTM model, as shown in Table 10, the attention layer was not suitable for use with an LSTM in our RNN model based on our parameter tuning results. Therefore, in the final test, we did not obtain attention scores for the RNN models.

In the detailed analysis in Figure 8, the GRU model’s comparatively high attention scores were widely distributed. However, the Elman RNN model’s high attention scores are located toward the front. These models’ high attention scores indicate the importance of the data sequence for classification. Therefore, in the Elman RNN model, the initial portion of the data was most important for classification.

Subject-independent evaluation is especially important in the domain of affective computing. Due to the small sample size, we could not split the data into three folds of training, validation, and testing, and instead we reported average scores of the repeated five-fold cross validation experiments. To compensate for this limitation, we further evaluated the performance of MLP models through the Leave-one-out cross-validation (LOOCV). The results are summarized in Table 13. Compared to the results in Table 11 and Table 13, the performances of MLP models measured using LOOCV and five-fold cross validation were not significantly different, and the results using LOOCV were slightly higher than the average scores in 1000 iterations of five-fold cross validation results. This could be because the training sample size increases in LOOCV compared to 5-fold cross validation.

Additionally, we investigated MLP models’ performance in binary classification scenarios such as happiness versus non-happiness. Table 14 shows the results using LOOCV in the MLP models. The binary classification performances were mostly higher than multi-class classification performance; however, the performances of testing peacefulness versus non-peacefulness were low because the boredom state was confused with the peacefulness state as in Table 12. Therefore, we tested a case using only peacefulness and happiness samples, which resulted in higher performance.

## 6. Discussion

In this study, we proposed two machine learning models to classify the emotions of AD patients: MLP-i and Elman RNN. From the accuracy aspect, the MLP-i model performed better than all the other CML or DL candidate models; however, it achieved high accuracy only when classifying happy and peaceful emotions; the boredom classification performance of this model was only 0.17%. Therefore, we suggest using the Elman RNN model to classify boredom. This model classified boredom with an accuracy of 44%; no better performance was achieved by any other model. Therefore, depending on the goals, future researchers should use caution when choosing among the suggested models.

As analyzed in Section 2.2.2, no previous studies have attempted to classify the emotions of AD patients using EEG signals (see Table 2). Kumfor et al. targeted bvFTD and SD patients and used patients’ GSR and facial data; however, from a medical aspect, those target patients differed from ours in terms of dementia type, age group, and nationality [40]. Furthermore, among the studies that targeted the emotional classification of patients with neurological disorders, no study trained RNN classification models or selected peacefulness and boredom as target emotions (see Table 3). Thus, this study complements the work performed by previous studies.

The previous EEG-based emotion classification studies targeted neurological disorder patients using 15 or 30 electrodes on non-commercial EEG sensors (see Table 3) [36,37,38,41]. Furthermore, the studies that analyzed healthy people’s EEG data for classifying emotions used EEG sensors with four or more electrodes [6,7,8,9,10,11,12,13,14,15,16,17,18,19,20,21,22,24,25,26,27,28,29,31,75]. However, in this study, as described in Section 3.4, we used EEG data from only two electrodes due to the practical restrictions set by the requirements of our project. We acknowledge that this may raise concerns regarding whether two electrodes are enough to provide sufficient quality of EEG data for emotion classification. In our previous studies, however, we analyzed EEG data from two electrodes and secured classification models with reliable performance [33,34]. Furthermore, Takahashi [23] and Shen et al. [25] also successfully used EEG data from two electrodes attached to the frontal area of the participant’s head for emotional classification. Additionally, in our investigation of the literature, studies that used the Muse Brainband for psychological and mental research exist [91]. Thus, as the results of previous studies and the performance of the proposed model indicate, the use of EEG data from only two electrodes can be used to classify emotions.

In this study, we adopted the EEG-based emotion classification framework of Seo et al.’s study [34], which was the latest state-of-the-art method in our literature review. As summarized in Table 1, most previous emotion classification studies employed traditional machine learning methods such as SVM, KNN, or MLP. By considering all these methods and more recent RNN models, we did a comprehensive optimization to find the best methodological setup for our problem. To further investigate how the results of the latest emotion classification for healthy people could be compared to our results on AD patients, we applied the MLP model to our dataset in a binary classification setting as in [34]. The results are summarized in Table 14. Although the used dataset and emotion settings were different, it can be seen that the current MLP model in the AD patients group achieved overall higher accuracies than the highest accuracy of 77.04% in [34] among those studies for healthy individuals in Table 1. Therefore, it implies that emotion classification model for healthy people can be applied for AD patients as well. To compare the two groups more systematically, however, additional studies should be conducted with data collected under the same setting.

A general approach of training models in machine learning divides the data into three datasets (training, testing, and validation). An essential condition for dividing the data is securing a large enough volume of data. However, in some studies that used human physiological data, practical limitations made it hard to collect enough data. Therefore, many studies adopted k-fold cross-validation or LOOCV approaches for validating their models’ performance [6,13,15,17,18,22,23,24,29,33,34,36,37,38,59,74,92,93]. Although this validation approach is not as robust as dividing the data into multiple sets, it has been recognized by the scientific community as a suitable validation approach. In our future study, we plan to collect more data from AD patients and evaluate our model on them.

In our data collection protocol, we requested that the participants estimate the strength of the target emotion using a four-point scale after showing each emotion-evoking video. The reason for interrupting the sequence of emotion-evoking videos with a question was the concern that AD patients with dementia might not remember their emotional status for each stimulus were the questions asked at the end of the experiment. However, for cross-checking the correlation between the target emotion and the subjective measurement, an ideal method would be to ask the participant at the end of the experiment which of the three emotions they felt for each stimulus. In our future work, we will reflect on this limitation.

Regarding the reason why the MLP models performed better than the RNN models, we hypothesize that the limited number of samples affected the RNN models’ performance. Deep learning models typically require a large number of samples to train models well. In this study, we used 60 samples for training and testing. Compared to the public EEG dataset, MindBigData contains 1,207,293 samples [94]. Using that dataset, Kim et al. and Gao et al. achieved accuracies of 95.95% and just under 95% for models that classified users viewing digits or other objects, respectively [95,96]. In this study, we investigated the possibility of using deep learning for classifying emotion using small numbers of EEG samples. Our current Elman RNN model did not reach the performance levels achieved by the MLP-i model; however, we expect that if the models were trained on a larger dataset or by using other DL approaches, this trend would change.

We also analyzed the attention scores of the GRU and Elmen RNN models in Section 5.4; however, we did not assign a high value to the GRU model’s attention score result because the performance of the GRU model was worse than that of the other CML and RNN models; therefore, analyzing this model’s attention score would not have a relevant impact. However, the Elman RNN model’s performance was the best among the RNN models. Additionally, for boredom classification, the model performed the best among all the tested models. Thus, analyzing the attention score of this model may be valuable.

Regarding the attention score analysis result for the Elman RNN model, the attention scores of the first 100 sequences were higher than those of later sequences. Considering the sampling rate (10 Hz), the first 10 s of EEG data after the stimulation began had a larger impact on the classification result. Therefore, we suspect that the emotional states of AD patients could be determined within 10 s after a stimulus. However, the underlying reasons regarding why 10 s of data had an influence on the Elman RNN model classification should be investigated in a future study.

In this study, we did not investigate the correlations between the emotion detection results and the AD patients’ MMSE points because the participants were unevenly distributed across the MMSE categories; thus, the number of participants in some of the MMSE categories was not large enough. In future work, we plan to invite more AD patients and investigate whether the MMSE score has any influence on the emotion detection results.

Furthermore, some studies have analyzed face processing in the brains of participants with Asperger syndrome [97,98], but to the best of our knowledge, EEG-based emotion detection for this group has not been explored. Our future study will investigate EEG-based emotion detection with participants having other types of disorders, such as autism and AD.

The limitations of this study are as follows. First, we used only a relatively small number of samples for training. Therefore, the generalizability of our results would need further validation. Second, we collected data from Alzheimer’s disease patients with dementia, all of whom were Korean women. Thus, applying our models or protocol to AD patients representing other cultural groups, genders, age groups, or types of dementia disease may lead to different results. Third, due to a relatively small dataset, we used a k-means cross validation instead of a train-test split. Although the former approach has been widely used, it is regarded as less robust than the latter. In future studies, we will address these limitations by collecting more data from more diverse groups of patients across different cultures, by using different emotion-evoking stimuli, and by investigating additional training techniques, such as using a CNN to develop a unified emotion classification model.

## 7. Conclusions

In this study, we developed classification models that can detect the emotions of AD patients using EEG signals as a data source. We achieved this result by collecting EEG data from 30 Korean patients with Alzheimer’s disease who were patients at a medical rehabilitation center. Moreover, we trained models using CML algorithms and RNNs. The accuracy measurements indicate that the MLP-i model achieved an average accuracy of 70.97% for the three target emotions, whereas the Elman RNN model reached an average accuracy of only 48.18% for the three target emotions. These models showed different strengths for classifying specific emotions: happiness and peacefulness for MLP-i and boredom for Elman RNN). Although we did not produce a unified emotion detection model for general emotion detection usage, considering that previous studies have mainly focused on patients with neurological disorders, our results open a new stream of research in the field of detecting the emotions of patients with AD. Based on our results, future caregivers and system developers can expect to obtain the emotional states of AD patients and utilize that information to provide better care. Additionally, in our future study, we will investigate how to perform near-real-time adaptation of VR content in our VR system with regard to emotions experienced by AD patients. We will design and implement the hardware and software infrastructure for responding as such.

## Figures and Tables

**Table 1:** Emotion classification of healthy people based on EEG data (up to four electrodes).

Study	Target Emotions	Approach	Accuracy (%)	Validation
[23]	Joy, anger, sadness, fear, relaxation	Support vector machine (SVM)	41.68	LOOCV
[25]	Engagement, confusion, boredom, hopefulness	SVM, k-nearest neighbors (KNN)	67.1 (SVM) 62.5 (KNN)	-
[7]	Sadness, disgust	Linear SVM	78.04 (Sad) 76.31 (Disgust)	-
[11]	Arousal, valence	SVM, K-means	-	-
[33]	Boredom, non-boredom	KNN	86.73	5-fold cross-validation
[34]	Boredom, non-boredom	Multilayer Perceptron (MLP)	79.98	5-fold cross-validation
[30]	Boredom, frustration	Analysis	-	-

**Table 2:** Studies by participant types.

Study	Health Status
[36,37,38]	Parkinson’s disease
[41]	Vegetative state(VS) and minimally conscious state(MCS)
[40]	Behavioural-variant FTD (bvFTD) and semantic dementia (SD)

**Table 3:** Studies on participants with neurological disorders.

Study	Physiological Data Source	Target Emotions	Approach	Validation
[36]	EEG (16 electrodes)	Happiness, Sadness, Fear, Anger, Surprise, and Disgust	SVM and Fuzzy KNN	10-fold cross-validation
[37]	EEG (16 electrodes)	Happiness, Sadness, Fear, Anger, Surprise, and Disgust	SVM and KNN	10-fold cross-validation
[38]	EEG (16 electrodes)	Happiness, Sadness, Fear, Anger, Surprise, and Disgust	SVM	10-fold cross-validation
[40]	GSR and facial	Positive, Neutral, and Negative	ANOVA	-
[41]	EEG (30 electrodes)	Happiness, and Sadness	SVM	Train test split

**Table 4:** MMSE-K results of the participants.

MMSE Range	Number of Participants
≥24	1
20–23	6
≤ 19	21
Unknown	2

**Table 5:** Tested classifiers.

Classifier	Option	Classifier	Option	Classifier	Option
IBk	No	LibSVM	Linear	J48	Default
	1/distance		Polynomial	JRip	
	1-distance		Radial	Naive Bayes	
Multilayer Perceptron (MLP)	t		Sigmoid	KStar	
	i	Decision Stump	Default	LMT	
	a	Decision Table		PART	
	o	Hoeffding Tree		Logistic	
	t,a	Random Tree		Simple Logistic	
	t,a,o	Random Forest (RF)		Zero R	
	t,i,a,o	REP Tree		One R	
**Parameter options of MLP for designing network (number of node per each layer)** “i”= number of features, “o” = number of labels, “t” = “i” + “o”; “a” = “t”/2					

**Table 6:** Interview results and label distribution.

	Interview	Result	Label	
Boredom	Others	5	Others	21
	No	16		
	Yes	6	Boredom	9
	Very Much	3		
Happy	Others	3	Others	5
	No	2		
	Yes	16	Happy	25
	Very Much	9		
Peaceful	Others	2	Others	4
	No	2		
	Yes	16	Peaceful	26
	Very Much	10		

**Table 7:** Initial test results of CML algorithms (top ten models).

Trained Algorithm	Window Size (s)	Option	Average Accuracy (%)	
			Before WSE	After WSE
MLP	80	i	37.33	68.83
MLP	80	a	38.50	68.00
MLP	80	t	36.67	66.50
MLP	80	t,a	40.83	64.83
MLP	50	t,a	40.67	64.17
Kstar	80	Default	40.67	64.17
PART	50	Default	41.67	64.00
MLP	80	o	43.83	63.83
Kstar	20	Default	31.67	63.67
RF	80	Default	45.67	63.33

**Table 8:** WSE results by algorithm and its option.

Trained Algorithm	Selected Features	Related Electrode		Related Frequency Band
MLP-a	AF8 Alpha DE	-	AF8	Alpha
	Gamma DASM	AF7	AF8	Gamma
	Gamma RASM	AF7	AF8	Gamma
MLP-i	AF8 Alpha DE	-	AF8	Alpha
	Gamma DASM	AF7	AF8	Gamma
	Gamma RASM	AF7	AF8	Gamma
MLP-t	AF7 Beta ABP mean	AF7	-	Beta
	AF8 Alpha DE	-	AF8	Alpha
	Gamma RASM	AF7	AF8	Gamma

**Table 9:** MLP parameter tuning results.

Trained Algorithm	Learning Rate	Epochs
MLP-a	0.811	105
MLP-i	0.991	256
MLP-t	0.470	229

**Table 10:** RNN model’s parameter tuning results.

r	m	b	d	a	bi	n	Epochs
GRU	2	Bi	256	True	-	-	487
LSTM	1	Single	512	False	-	-	656
Elman RNN	3	Single	512	True	False	tanh	424

**Table 11:** Final test results—1000 iterations of five-fold cross-validation.

	Accuracy (%)			AUC			F1-Score		
	Average	Min	Max	Average	Min	Max	Macro	Weight	Micro
MLP-a	67.18	53.33	76.67	0.699	0.605	0.765	0.486	0.619	0.672
MLP-i	70.97	61.67	76.67	0.697	0.630	0.750	0.516	0.657	0.710
MLP-t	63.74	55.00	71.67	0.642	0.577	0.701	0.464	0.592	0.637
RNN - GRU	42.83	33.33	53.33	0.523	0.427	0.630	0.377	0.418	0.428
RNN - LSTM	44.58	35.00	53.33	0.513	0.429	0.606	0.311	0.395	0.446
RNN - Elman RNN	48.18	35.00	61.67	0.592	0.439	0.721	0.483	0.482	0.482

**Table 12:** Confusion matrix of the final test (MLP models—1000 iterations|RNN models—100 iterations).

**MLP-a**				**GRU**			
B	H	P	← Classified as	B	H	P	← Classified as
0	1272	7728	B	139	276	485	B
0	17573	7427	H	29	1169	1302	H
29	3238	22733	P	141	1197	1262	P
**MLP-i**				**LSTM**			
B	H	P	← Classified as	B	H	P	← Classified as
15	1326	7659	B	4	210	686	B
39	19325	5636	H	21	785	1694	H
252	2506	23242	P	12	702	1886	P
**MLP-t**				**Elman RNN**			
B	H	P	← Classified as	B	H	P	← Classified as
0	2299	6701	B	396	279	225	B
187	17183	7630	H	157	1203	1140	H
834	4103	21063	P	166	1142	1292	P
B: Boredom, H: happiness, P: Peacefulness							

**Table 13:** LOOCV results for MLP models.

	Accuracy (%)	Weighted F1-Score
MLP-a	68.33	0.631
MLP-i	71.67	0.662
MLP-t	65	0.603

**Table 14:** LOOCV results for MLP models in binary classification.

	MLP-i		MLP-a		MLP-t	
	Accuracy (%)	Weighted F1	Accuracy (%)	Weighted F1	Accuracy (%)	Weighted F1
B - NB	85	0.781	85	0.781	83.33	0.833
H-NH	83.33	0.831	73.33	0.726	78.33	0.784
P-NP	58.33	0.585	51.67	0.518	60	0.601
P-H	84.31	0.843	80.39	0.801	76.47	0.761
B: Boredom, NB: Non-boredom, P: Peacefulness NP: Non-peacefulness, H: happiness, NH: Non-happiness						
